# Permanent lesion in rostral ventromedial medulla potentiates swim stress-induced analgesia in formalin test

**Published:** 2014-03

**Authors:** Ali Shamsizadeh, Neda Soliemani, Mohammad Mohammad-Zadeh, Hassan Azhdari-Zarmehri

**Affiliations:** 1 Physiology-Pharmacology Research Center, Rafsanjan University of Medical Sciences, Rafsanjan, Iran; 2 Department of Physiology & Pharmacology, Cellular and Molecular Research Center, Sabzevar University of Medical Sciences, Sabzevar, Iran; 3 Department of Basic Sciences, Torbat Heydariyeh University of Medical Sciences, Torbat Heydariyeh, Iran

**Keywords:** Analgesia, Formalin test, Inactivation, Lesion, Rostral ventromedial medulla, Swim stress

## Abstract

***Objective(s):*** There are many reports about the role of rostral ventromedial medulla (RVM) in modulating stress-induced analgesia (SIA). In the previous study we demonstrated that temporal inactivation of RVM by lidocaine potentiated stress-induced analgesia. In this study, we investigated the effect of permanent lesion of the RVM on SIA by using formalin test as a model of acute inflammatory pain.

***Materials and Methods:*** Three sets of experiments were conducted: (1) Application of stress protocol (2) Formalin injection after exposing the animals to the swim stress (3) Either the relevant vehicle or dopamine receptor 1 (D1) agonist R-SKF38393 was injected into the RVM to cause a lesion. For permanent lesion of RVM, R-SKF38393 was injected into the RVM. Forced swim stress in water was employed in adult male rats. Nociceptive responses were measured by formalin test (50µl injection of formalin 2% subcutaneously into hind paw) and pain related behaviors were monitored for 90 min.

***Results:*** In the unstressed rats, permanent lesion of the RVM by R-SKF38393 decreased formalin-induced nociceptive behaviors in phase 1, while in stressed rats, injection of R-SKF38393 into the RVM potentiated swim stress-induced antinociception in phase 1 and interphase, phase 2A of formalin test. Furthermore, R-SKF38393 had pronociceptive effects in phase2B whereas injections of R-SKF38393 resulted in significant difference in nociceptive bahaviours in all phases of formalin test (*P*<0.05).

***Conclusion:*** The result of the present study demonstrated that permanent inactivation of RVM can potentiate stress-induced analgesia in formalin test.

## Introduction

Although stress-induced analgesia (SIA) is a fundamental pain inhibition response that happens after exposure to a stressful situation, stress can as well enhance sensitivity to pain, referred to as “stress-induced hyperalgesia”. Pain facilitation and inhibition is mediated by brainstem pain- modulaing system. The current experiment investigated the importance of inhibitory control from the rostral ventromedial medulla (RVM) system. Experimental animal models of this phenomena help clarify the basic mechanisms of nociception as well as find novel therapeutic agents for disorders related to pain and stress (1). 

Stress has been shown to cause stress-induced analgesia that is activated by endogenous pain inhibitory systems (2-6). In some stressful situations, the blockade of the endogenous opioid system did not reverse SIA and this supports the idea that non-opioid mechanisms may be involved in SIA (7, 8). 

Depending on the features of a stressor (such as duration, intensity, and temporal aspects of the same stressor), the nature of the analgesic response might be different (9, 10).

The anatomical regions involved in pain modulation comprise cortex, hypothalamus, and brain stem including periaqueductal gray (PAG) matter, RVM and dorsal horn of the spinal cord (11, 12). Several studies have shown that the PAG-RVM modulates opioid analgesia (11). It has been revealed that the RVM is involved in top-down pain-modulation (13-15). Thus, RVM might be directly or/and indirectly (projection from PAG) an important site of action for SIA as supraspinalantinociception (1).

Electrophysiological experiments have discovered three types of neurons in the RVM: on-cell, off-cell and neutral cell. Electrical excitation of the on-cells elicits facilitatory influence on pain processing. Conversely, electrical excitation of off- cells results in pain-inhibitory effect. The neutral cells do not appear to contribute to nociception. Thus, the RVM has a facilitatory and inhibitory role in pain control (16). 

The formalin test can show biphasic nociceptive responses; phase 1 is caused by peripheral stimulation (17, 18), and phase 2 evokes an inflammatory pain that is produced by hyper excitability of the spinal cord neurons (19).

The present study investigates the role of RVM in stress-induced analgesia through nociceptive behavior due to injection of formalin. There are few studies examining the role of PAG in stress-induced analgesia (20). Projections of the PAG to the RVM, which in turn innervate the dorsal horn of the spinal cord might mediate the antinociceptive capabilities of the PAG and underlie PAG-mediation of SIA (21). In the previous study we demonstrated that temporal inactivation of RVM by lidocaine potentiated stress-induced analgesia (22, 23).Accordingly, we decided to destroy RVM to understand more about the function of RVM in swim stress-induced analgesia.

## Materials and Methods

***Subjects***

**Chart 1:**
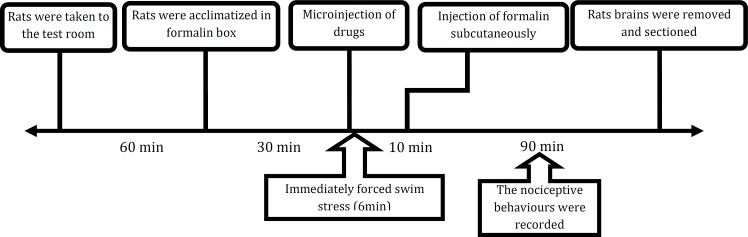
Timeline of the experimental procedures

Wistar rats (220–300 g) were purchased from Razi Institute (Karaj, Iran). Animals were housed in groups of three rats per cage at temperature-controlled room, under a 12 hr light-dark cycle with lights on from 7:00 to 19:00. Food and water were provided. All experiments were done in accordance with the National Institutes of Health Guide for the Care and Use of Laboratory Animals (NIH Publication No. 80-23, revised 1996) and were approved by the Research and Ethics Committee of Rafsanjan University of Medical Sciences, Rafsanjan, Iran.Three sets of experiments were conducted: (1) Application of stress protocol (swim stress test, 6 min at 20±1˚C). In this set of experiments, after exposing the animals to the swim stress, rats were received formalin subcutaneously (2). Formalin injection after exposing the animals to the swim stress (3). Either the relevant vehicle or R-SKF38393alone was microinjected into RVM followed by formalin injection after exposing the animals to the swim stress (Chart 1).

***Drugs***

Two percent formalin (formaldehyde, Temad, Iran) was prepared in sterile physiological saline solution (Soha, Iran), and R-SKF38393 was dissolved in saline as well. 

***General procedure***

Rats were initially anaesthetized with ketamine (100 mg/kg) and xylazine (10 mg/kg) and afterward a 23-gauge, 3 mm-long stainless steel guide cannula was stereotaxically(24) lowered 2 mm above the RVM by applying coordinates from the atlas of Paxinos and Watson(25): incisor bar -3.3 mm, 10.5-11 mm posterior to the bregma, midline to the sagittal suture and 10.6 mm down from top of the skull. Direct intra-RVM administration of drugs or the respective vehicle was performed by implanting the guide cannula 7 days before the experiments. The cannula was anchored with dental cement to stainless steel screws in the skull. Immediately after waking up from the surgery, rats were returned to their home cages. On the day of the experiment, rats were transferred to individual experimental room and allowed to acclimatize for 60 min before drug injection. Direct intra-RVM administration of drug, or respective vehicle was conducted with a stainless steel cannulae (30-gauge; 0.3 mm outer diameter) connected through a polyethylene tube to a Hamilton syringe, inserted through the guide cannula and extended 2 mm beyond the tip of the guide cannula to reach the RVM. A volume of 0.5 μl of drug or vehicle was injected into the RVM over a period of 60 sec and the injecting cannula was gently removed 1 min later. After performing stress procedures (6 min in swim stress) and drying the animals just in the swim stress model, formalin was injected into the plantar surface of right hind paw using a disposable insulin syringe with a fixed 30-gauge needle (26).

***Swim stress test procedure***

Rats were initially transferred to the test room and let to acclimatize for 60 min before the commencement of the experiment. After reduction in environmental stress, swim stress tests were performed immediately after injections of either drug or vehicle. Two rats were placed in a plastic pool (50 cm high) filled with water maintained at 20±1˚C for 6 min. Animals were thoroughly dried and then formalin was injected into the plantar surface of right hind paw. All experiments were carried out daily between 8 am and 4 pm (27-29). 

***Formalin test***

Rats were moved to the test room at least 1 hr before starting the experiment. Formalin tests were performed in clear plastic boxes (30×30×30 cm) with a mirror being placed underneath at a 45° angle to allow an unimpeded view of the animals’ paws. In the present study, rats were first acclimatized for 30 min in an acrylic observation chamber and then formalin (50 µl; 2%) was injected subcutaneously into the plantar surface of the right hind paw using a 30-gauge needle. To ensure stable scores from formalin, it was necessary to certify that the needle was inserted 5 mm under the skin. Each rat was then immediately returned to the observation box, and behavioural recording was performed. Pain behaviours were scored as follows: 0, the injected paw was not favoured; 1, the injected paw had little or no weight placed on it; 2, the injected paw was elevated and not in contact with any surface; and 3, the injected paw was licking or biting. Recording of the nociceptive behaviors was performed immediately after formalin injection (time 0) and continued for 60 min. The score obtained from nociceptive behaviours for each 3-min intervals was calculated as weighted average of the number of seconds spent ineach behaviour, from the start of the experiment. The scores were recorded in normal rats as well as in those exposed to swim stress test. In each group, the behavioural responses of each rat during the first phase (1-7 min) inter-phase (8-14 min) and the second phase (15-90 min) was separately evaluated. In order to induce the RVM lesion in this study, dopamine receptor 1 (D1) agonist R-SKF38393 (SKF) was injected on the day of surgery (26).

***Histology***

By the end of the experiments, rats were deeply anaesthetized with an overdose of ketamine followed by injecting a volume of 0.5 μl of pontamine sky blue (0.2%) into the cannula site. Afterward, rats were transcardially perfused with 100 ml of 4% formalin solution and the brain was removed and sectioned.

Rats with microinjection and diffusion sites being located within the RVM were exclusively included in the results. 

***Statistics***

Data are presented as mean ± SEM. The formalin pain score in all groups were subjected to one-way ANOVA followed by protected Dunnett/Newman-Keuls tests for multiple comparisons as needed. The first phase (1–7 min), interphase (8–14 min), and second phase (15-90 min) of the formalin test were analyzed separately while using one time point for each phase: a time course of 7 min for phase 1 and interphase, and 75-min duration for phase 2. The defined level for statistical significance was *P*-value<0.05.

## Results

***Effects of intra-RVM microinjection of R-SKF38393 alone on formalin-induced nociceptive behaviours***

**Figure 1:**
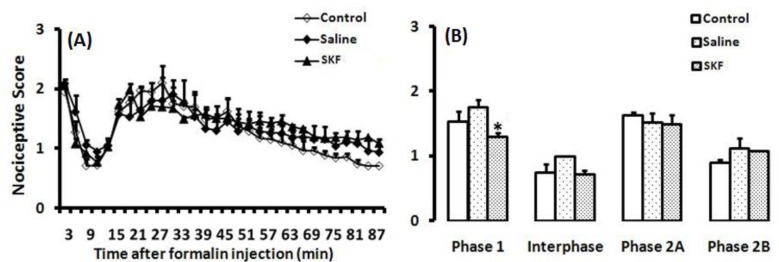
Time scores of nociceptive behaviours induced by formalin (mean ±SEM of 9-10 rats per group) measured every 3 min for 90 min (A) and bar chart for formalin test in control, saline and R-SKF38393 groups (B). The columns represent the mean of nociceptive score in each phase: phase 1 (min 1–7), interphase (min 8–14) and phase 2A (min 15–60) and phase 2B (min 61–90), (B). * *P*<0.05 in comparison with control group

In the control group, that received no swim stress, formalin injection into the hind paw induced typical biphasic pain response. The first and second phases were separated by a quiescent interphase, which is a characteristic of formalin test (Figure 1).

**Figure 2:**
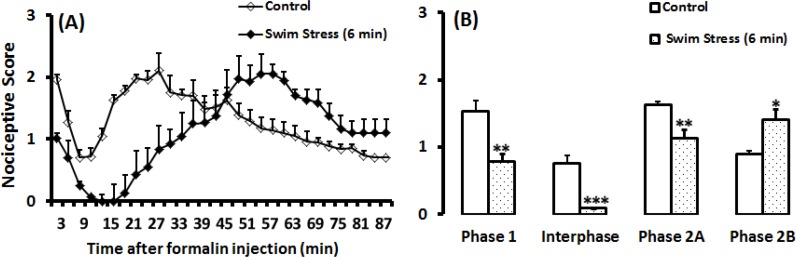
Time scores of nociceptive behaviours induced by formalin (mean ±SEM of 9-10 rats per group) following swim stress measured every 3 min for 90 min (A) and bar chart for formalin test after swim stress in 50-cm high water and control group (B). The columns represent the mean of nociceptive score in each phase: phase 1 (min 1–7), interphase (min 8–14) and phase 2A (min 15–60) and phase 2B (min 61–90), (B). * *P*<0.05; ** *P*<0.01 and *** *P*<0.001 in comparison with control group

† *P*<0.01 compared to stress-respective-control group

The intra-RVM injection of R-SKF38393 reduced pain in the first phase of formalin test [F (2, 23)=2.360; *P*=0.031; (Figure 1B)],but it had no effect on the interphase or second Phase: for interphase [F (2, 23)=0.680; *P*=0.517; (Figure 1B)], and phase 2A [F (2, 23)=0.778; *P*=0.471; (Figure 1B)], and had pronociceptive effect on phase 2B [F (2, 23)=2.859; *P*=0.078; (Figure 1B)].

***Effects Swim stress on nociceptive behaviors of formalin test***

Swim stress potentiated the antinociceptive response in phase 1 [T(1, 14)=2.90; *P*=0.011; (Figure 2B)], interphase [T (1, 14)=3.214; *P*=0.001; (Figure 2B)], and phase 2A [T (1, 14)=3.074; *P*=0.008; (Figure 2B)], and had pronociceptive effect on phase 2B [T (1, 14)=2.751; *P*=0.021; (Figure 2B)].

***Effects of RVM lesion induced by SKF on antinociceptive behaviors of swim stress in formalin test***

**Figure 3:**
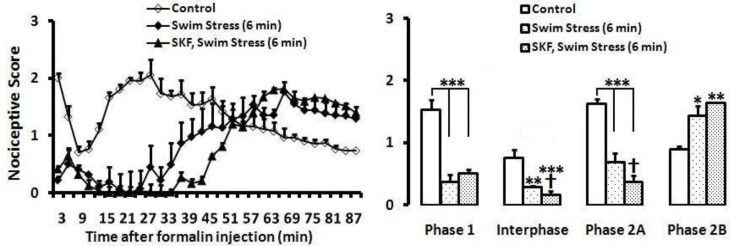
Time scores of nociceptive behaviours induced by formalin (mean ±SEM of 9-10 rats per group) following swim stress or SKF/swim stress measured every 3 min for 90 min (A) and bar chart for formalin test after swim stress and control group (B). The columns represent the mean of nociceptive score in each phase: phase 1 (min1–7), interphase (min 8–14) and phase 2A (min 15–60) and phase 2B (min 61–90), (B). * *P*<0.05; ** *P*<0.01 and *** *P*<0.001 in comparison with control group

† *P*<0.01 compared to stress-respective-control group

Following RVM lesion induced by SKF, swim stress potentiated the antinociceptive response in phase 1 [F (2, 26)=18.735; *P*=0.000; (Figure 3B)], interphase [F (2, 26)=6.687; *P*=0.005; (Figure 3B)], and phase 2A [F (2, 26)=42.397; *P*=0.000; (Figure 3B)], and had pronociceptive effect on phase 2B [F (2, 26)=18.727; *P*=0.000; (Figure 3B)].

## Discussion

It has been argued that blocking the RVM could illustrate the role of RVM in pain modulation. In the previous study we demonstrated that temporal inactivation of RVM by lidocaine potentiated stress-induced analgesia (22, 23). Total lesion induced by SKF38393 microinjection was consistent with temporary inactivation.Lidocaine blocks a particular neuronal area through limiting Na currents and elimination of lidocaine takes upwards of 2 hr, (30) therefore we were used it during 90 min of formalin test (31).

The dopamine receptor antagonist (D1) SKF38393 induces neurotoxic effects as observed in histological studies (32-34). Explaining the differences between temporary and total inactivation manipulation, Manning utilized NADM-induced lesion as an excitotoxin-induced lesion to completely and unilaterally inactivate the central amygdale to address dissociation of locomotor and antinociceptive effects of morphine. Excitotoxin-induced lesion was chosen over temporary inactivation to generalize different doses of morphine. Furthermore, it makes valid comparisons between data (35). In another study, temporary inactivation of RVM by lidocaine and previous permanent inactivation by ibotenic acid supported that neorotensin and NADM receptor in RVM contribute to descending facilitator influences in secondary hyperalgesia (36).

In the current study, permanent inactivation of RVM enhanced analgesia responses as a result of stress. The RVM-PAG pain modulating system involved a supraspinal center in processing pain. Due to a large number of opioid receptors, PAG participates in antinociceptive responses (16). In fact, PAG responses manipulate the RVM and then project through dorsal longitudinal fasciculus (DLF) to dorsal horn of spinal cord (37). The RVM, including the nucleus raphe magnus and adjacent reticular nuclei, plays pivotal role in top-down pain modulation system (37, 38). Physiological characterization of neurons in the RVM suggests calling them on-cells, off-cells and neutral cells (16). Electrical excitation of on-cells elicits facilitatory influence on pain processing and it is reported that the firing rate of on-cells decreases or inhibits by µ-opioid agonists (39, 40). On the other hand, electrical excitation of the off-cells causes pain-inhibitory influence while the pause of firing activity in off-cells increases with morphine (13, 41, 42). Based on several reports, the neutral-cells show no change in firing rate before nociceptive reflex and fail to response to opioids (13, 41, 42). Some research state that the RVM contributes to stress-induced analgesia or hyperalgesia as a phenomenon in pain modulation (43-48). Watkins *et al* in 1983 demonstrated that it is the origin of the analgesia via descending pathways lying solely within the dorsolateral funiculus of the spinal cord which mediates front paw analgesia by foot shock and classically-conditioned analgesia (49). Evidences for bidirectional role of RVM in pain control develop an idea that potentiated analgesia due to stress is the result of facilitatory role of RVM. In a previous study, Coderre *et al *reported that RVM akin to positive feedback loop is activated in exposure to noxious stimulations (50). Moreover, in male rats receiving electrocupuncure to the tail, immediate early gene, C-fos, in the RVM was expressed more than control; thus indicating that RVM is activated during noxious conditions (21). Being consistent with our study, injection of methysergide (as a serotonergic antagonist) into the RVM markedly potentiated continuous cold-water swims, but not intermittent cold-water swims analgesia (51). Similar to the current study, diazepam potentiated cold swim stress analgesia (52) as well as an antagonist of nitric oxide synthase selectively augmented swim stress analgesia in rats (53). 

On the contrary, in some related studies, a pain-inhibiting role was identified for RVM. For instance, activation of RVM was required for opioid analgesia (54). There is evidence indicating that male rats were restraint while the RVM had been inactivated with lidocaine, as well as having a decreased tail-flick latency (55). This conclusion is in contrast with our finding. Therefore, differences in pain and stress models must be considered. 

As being known, the RVM is required to facilitate pain associated with inflammation or prolonged noxious stimulus (-). Moreover, it has been reported that subcutaneous injection of formalin can increase the ongoing activity of on-cells in RVM (60). Different studies suggest that inflammation due to mustard oil leads to strongly activating the on-cells and nearly suppressing the off-cells in RVM (57). Our results appear to confirm the previous studies; hence, inactivation of the RVM during formalin test, as in persistent pain, leads to potentiate the stress-induced analgesia as a result of on-cells inactivation in RVM. Electrophysiology studies into on-cells during formalin injection would complete this evidence. It is thus reasonable to considering neurotransmitters such as GABA and Glutamate due to evidences suggesting GABAergic and Glutamatergic signaling contribution to SIA (1) and modulated interphase of formalin test (61). GABA receptor mediated inhibition of the off-cells in RVM and Glutamate receptor is tied to facilitating pain in the on-cells in RVM (16, 62, 63). Recently, some neurotransmitter and neuropeptide such as orexin have been recognized that are involved in SIA being likely to clarify the function of RVM in pain modulation like SIA (29, 64).

## Conclusion

The present experiment demonstrated the role of RVM in pain suppression upon exposure to stressful situation. Hence, inactivation of the RVM, permanently by SKF38393, could exaggerate stress-induced analgesia.
